# Triple-Type Feature Extraction for Palmprint Recognition

**DOI:** 10.3390/s21144896

**Published:** 2021-07-19

**Authors:** Lian Wu, Yong Xu, Zhongwei Cui, Yu Zuo, Shuping Zhao, Lunke Fei

**Affiliations:** 1 School of Mathematics and Big Data, Guizhou Education University, Guiyang 550018, China; zhongweicui@gznc.edu.cn (Z.C.); zuoyu@gznc.edu.cn (Y.Z.); 2 Bio-Computing Research Center, Harbin Institute of Technology, Shenzhen 518055, China; laterfall@hit.edu.cn; 3 School of Computer, Guangdong University of Technology, Guangzhou 510006, China; yb77458@connect.um.edu.mo

**Keywords:** biometrics, palmprint recognition, triple-type feature descriptors, matching score fusion

## Abstract

Palmprint recognition has received tremendous research interests due to its outstanding user-friendliness such as non-invasive and good hygiene properties. Most recent palmprint recognition studies such as deep-learning methods usually learn discriminative features from palmprint images, which usually require a large number of labeled samples to achieve a reasonable good recognition performance. However, palmprint images are usually limited because it is relative difficult to collect enough palmprint samples, making most existing deep-learning-based methods ineffective. In this paper, we propose a heuristic palmprint recognition method by extracting triple types of palmprint features without requiring any training samples. We first extract the most important inherent features of a palmprint, including the texture, gradient and direction features, and encode them into triple-type feature codes. Then, we use the block-wise histograms of the triple-type feature codes to form the triple feature descriptors for palmprint representation. Finally, we employ a weighted matching-score level fusion to calculate the similarity between two compared palmprint images of triple-type feature descriptors for palmprint recognition. Extensive experimental results on the three widely used palmprint databases clearly show the promising effectiveness of the proposed method.

## 1. Introduction

As one of the most important solutions for performing personal authentication our modern society, biometric recognition can effectively and efficiently identify an individual based on one’s physiological or behavioral traits [1,2,3]. There have been various biometric recognition technologies such as face, fingerprint and gait recognition technologies, which has been successfully used for many practical applications such as mobile payment, electronic control and security checking [4,5,6]. In recent years, as a relatively new emerging biometric technology, palmprint recognition has received tremendous research interest, because it contains many discriminative and reliable features such as principal lines and rich textures [7,8,9]. Moreover, palmprint recognition is a non-invasive and hygienic biometric technology [10,11], which make people prefer to use palmprint for personal authentication, especially in the current outbreak of COVID-19. Therefore, a growing number of studies turn to the important and challenging palmprint recognition technology [12,13,14,15,16].

There have been many palmprint recognition methods proposed in the past decades, which can be roughly classified into three categories according the types of palmprint images [17]: high-resolution palmprint [18], low-resolution palmprint [7] and three-dimensional (3D) palmprint recognition [19] methods. High-resolution palmprint recognition methods generally extract the ridge directions, ridge densities and the minutiae points for personal authentication for forensic applications [18]. 3D palmprint recognition mainly extracts the 3D surface features of palm surface such as curvature features. In general, both high-resolution palmprint and 3D palmprint images need to be captured with special-designed and expensive devices, which make them impractical for commercial and civil applications. For these reasons, more efforts were devoted to low-resolution palmprint recognition in recent years, where the low-resolution palmprint images can be easily captured by using the common image acquisition equipments such as mobile phone and normal camera. In this paper, we also focus on low-resolution palmprint feature extraction and recognition, and palmprint recognition of this paper refers to low-resolution palmprint recognition unless otherwise stated.

So far, there have been extensive methods proposed for palmprint recognition in the literature. The original palmprint recognition methods usually extract the visible texture-based feature-based features of palmprint such as local binary pattern (LBP) based feature descriptor [20], and the visible line feature-based methods such as the principal lines and wrinkles [21,22]. Most existing studies [17] have shown that the coding-based methods are one of the most effective palmprint representations. Due to these, a number of coding-based methods were proposed for palmprint recognition [23,24,25,26,27,28,29,30]. The original code method was proposed by Kong et al. [23], which encoded the Gabor filtering responses of palmprint images based on winner-take-all rule and achieved promising palmprint verification performance. Inspired by that, Guo et al. [24] proposed a binary orientation co-occurrence vector (BOCV) method by encoding the Gabor filtering responses on multiple directions. After that, Zhang et al. [25] extended the BOCV (EBOCV) method by filtering out the fragile bits of the BOCV codes. Further, Fei et al. [26] proposed a double orientation code (DOC) method by encoding the direction features of the top-two filtering responses. Zhang et al. [9] and Fei et al. [17] made two surveys surveys for the representative palmprint recognition methods. Moreover, the subspace learning, sparse representation and deep-learning were also successfully used for palmprint recognition. For example, Lu et al. [31] proposed eigenpalm method by extracting the PCA data of palmprint. Imad et al. [32,33] proposed a sparse representation-based method by extracting the subspace features such as PCA and LDA information and establishing an ensemble sparse representation. Svoboda et al. [34] comparatively studied the deep convolutional neural networks for palmprint recognition. Fei et al. [35] proposed a binary code learning method for heterogeneous palmprint recognition by learning the complementary features of multiple modalities of palmprint images. In addition, Genovese et al. [36] proposed a PalmNet method by applying the Gabor responses and PCA into the convolutional networks. In general, these learning-based methods usually require many labeled samples to learn and extract the discriminative features. For example, subspace-based methods require enough labeled samples to measure the within-class and between-class distances. Representation based methods need a plenty of labeled samples to completely represent the query samples, and the deep-learning methods require a massive training samples to fine-turn a plenty of parameters of the networks. Therefore, how to directly extract the discriminative features of palmprint images remains an interesting and important challenge.

**Figure 1:**
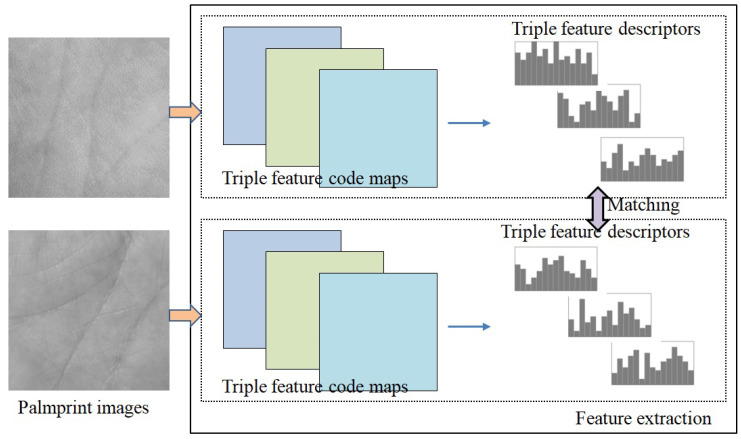
The basic idea of our proposed TFD method for palmprint recognition. We first extract and encode the triples types of features of a palmprint image, including the texture, gradient and direction features. Then, we form histogram-based feature vectors as the palmprint feature descriptors. Finally, we employ the weighted matching score fusion for feature matching and recognition.

In general, a palmprint image contains the principal lines, wrinkles and ridge patterns, which are the most important characteristics of a palmprint. Of them, the principal line and wrinkles has visible edges and thus they carry gradient-based features and direction-based features. In addition, ridge-based information of a palmprint forms the texture features. In other words, the direction-based, gradient-based and texture-based features are the most important and distinct features of a palmprint. Motivated by this, in this paper, we propose a triple-type feature descriptor (TFD) for palmprint representation and recognition. Figure 1 shows the basic idea of the proposed method. First, we extract the texture features by encoding the top-two maximum pixel distance within a local neighborhood, extract the gradient features by encoding the top-two edge responses of palmprint images, and further extract the direction features by encoding the most dominant direction and the most reliable direction. Then, to make the triple-features invariant to small misalignment, we calculate the block-wise histograms of the triple-type feature codes and cluster them into feature vectors, respectively, as the final feature descriptors of the palmprint. Finally, we employ the weighted matching score fusion to fuse triple-type feature for palmprint recognition. Extensive experimental results on three widely used contactless palmprint databases clearly demonstrate the effectiveness of the proposed method.

The main contribution of this paper can be summarized as follows:

- We propose a new palmprint descriptor by extracting triple-type inherent features of palmprint image. Unlike single-type feature descriptor, our proposed method can completely represent the multiple important and inherent characteristics of palmprint images.
- Unlike the recently learning-based methods which require many training samples, our proposed method can effectively extract the discriminative feature manually without requiring any training samples, such that our proposed method is suitable for the few-shot and even zero-shot biometric recognition tasks.
- We conduct both palmprint verification and palmprint identification experiments on three widely used challenging databases and the experimental results demonstrate that our proposed method consistently outperforms previous state-of-the-art methods.

The rest of this paper is organized as follows. Section 2 briefly the related topics. Section 3 elaborates our proposed triple-type feature descriptors for palmprint recognition. Section 4 presents the experimental results. Section 5 offers the concluding remarks.

## 2. Related Work

In this section, we first introduce the preprocessing of palmprint images. Then, we briefly review the representative palmprint feature extraction methods. Lastly, we introduce the fusion schemes of multiple biometrics.

### 2.1. Preprocessing of Palmprint Images

In general, the original palmprint images are captured from the whole hands, which usually consist of the all hand as well as the background images. Due to this, an original captured palmprint image needs to be preprocessed to crop the center part of the original palmrpint image. In other words, we need to extract the region of interest (ROI) of the original palmprint image before performing palmprint feature extraction and recognition [7]. So far, there have been several ROI extraction methods for palmprint image preprocessing, such as the PalmCode and Deep-learning methods [7,37,38]. Particularly, the PalmCode method [7] is one the most popular ROI extraction method. Specifically, it first detects the boundary of the whole palm from the original palmprint image by using thresholding principle. Then, it dominates the crossing valley points between fingers as the reference points, based on which a rectangular coordinate system can be established. After that, the center part of the palm image is further located based on the coordinate system, and cropped as the ROI of the palmprint image. In this paper, we use the PalmCode method to extract the ROIs of the palmprint images for the experiments.

### 2.2. Feature Extraction for Palmprint Representation

There have been a number of feature extraction methods for palmprint representation which can be roughly classified into two categories: heuristic feature representation and learning feature representation. The heuristic feature representation methods mainly extract the hand-crafted features of palmprint images such as the lines and direction features [39,40]. For example, Huang et al. [21] proposed a modified finite randon transform (MFRAT) method to extract the three principal lines of palmprint for personal verification. Wu et al. [22] proposed a DoG method to extract both principal lines and wrinkles for palmprint representation. In addition, Dai et al. [20] designed a multiple band wavelet-based method to extract the texture features of palmprint images. In recent years, more efforts were devoted into learning-based palmprint representation due to the impressive performance of deep learning on image classification [36,38]. For example, Genovese et al. [36] proposed a PalmNet method by using CNN for palmprint recognition. In addition, Fei et al. [38] proposed a binary code learning method to extract the discriminative features for palmprint recognition. Furthermore, Fei et al. [41] proposed a multi-feature learning method for palmprint recognition by learning the complementary binary codes of multiple types of features.

In general, the learning-based methods usually require enough labeled samples to training the feature extraction model. Due to this, there are still many studies focus on hand-crafted feature extraction of palmprint images, and the direction-based coding methods are one of the most effective hand-crafted feature representations [27]. The most typical direction coding methods are the dominant direction-based feature descriptors such as competitive code [23], discriminative and robust competitive code (DRCC) [39], and robust line orientation code (RLOC) [27] methods. They usually first define a series of feature extraction templates, such as Gabor filters and MFRAT, to convolve a palmprint image to compute the direction feature responses. Then, they treat the direction of the template that has the maximum convolution response as the dominant direction of the palmprint image and convert them into feature codes. For example, the competitive code method used six Gabor filters as the template and used the direction index of the Gabor filter producing the maximum filtering response as the direction features. Unlike the competitive code, the RLOC method used the MFRAT as the templates to extract the dominant direction features based on the same winner-take-all rule as the competitive code. The DRCC method improved the competitive code method by using the filtering results within a local region to extract the robust dominant direction features. Different from the dominant direction feature descriptors, there have also a number of methods that extract multiple direction features of palmprint images. For example, the BOCV method calculated the convolution responses between the direction feature templates and the palmprint image, and converted the convolution results on all directions into multiple feature codes for palmprint recognition. Moreover, the E-BOCV method filtered out the fragile bits with small direction responses from the BOCV code maps. In addition, the double orientation code method [26] useed twelve Gabor filters with different directions to convolve with a palmprint image. The two maximum responses of the two dominant directions were encoded into decimal codes. More direction feature extraction methods were comparatively studied in the palmprint survey literature [9,27].

### 2.3. Multiple Feature Fusion

It is a widely used and effective way that fuses multiple features to improve the overall biometric recognition performance. There have been four typical fusion schemes for multiple features fusion: sensor level, feature level, matching score level and decision level [42,43,44] fusion. In general, sensor level fusion scheme [42] uses different kinds of sensors such as different cameras to capture multiple samples of the same biometric trait, which are then fused by using other kinds of fusion schemes. Feature level fusion [44] mainly extracts different kinds of features by using different methods and then concatenates these features for representation and matching. In addition, decision level fusion scheme means that different decision makers such as different classification methods are used, based on which the final decision is made based on some strategy such as voting scheme. By contrast, score level fusion scheme is to first extract multiple features, and then to perform feature matching of them respectively. Finally, the matching results of different features are combined to calculate the overall matching result. So far, the score level fusion is one of the most widely used fusion scheme because it is a simple yet effective scheme in multiple feature fusion. In this paper, we use a weighted matching score level fusion to fuse the triple-type features of palmprint images for palmmprint recognition.

## 3. Triple-Type Feature Encoding and Matching

In this section, we first introduce the triple-type feature extraction of our proposed method. Then, we detail the matching fusion of the triple-type features.

### 3.1. Texture Feature Extraction of Palmprint Images

It is well recognized that texture is one of the most important characteristics of a palmprint image. Motivated by this, we first extract the texture features from palmprint images. Different most existing LBP-like texture descriptors [20], we propose a robust texture feature descriptor by selecting the neighbors with the maximum distances to the center point in the local 3×3 neighborhood. Specifically, we first calculate the absolute gray value differences between the center point and its eight neighbors, obtaining eight gray value differences: $d_{i}\left(i=1,2,\ldots ,8\right)$. Then, we sort these gray value differences in descending order. Third, we select the two points which has the two maximum gray value differences with the center point, and encode the direction number of them into texture feature code, as follows:

(1) $$Tcode=\left(m_{1}-1\right)\times 8+\left(m_{2}-1\right),$$

where Tcode represents the texture feature code of the center point of the palmprint image. $m_{1}$ and $m_{2}$ denotes direction numbers of the two neighbor points with the top-two gray value differences. Specifically, $m_{1}$ and $m_{2}$ can be calculated as follows:

(2) $$m_{1}=\arg\max_{j}\left\{d_{j}\right\},$$

and

(3) $$m_{2}=\arg\max_{j}\left\{\left\{d_{j}\right\}-d_{m_{1}}\right\}$$

**Figure 2:**
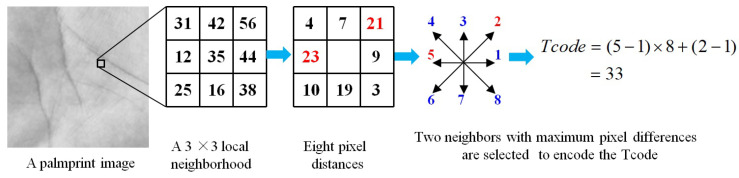
The basic procedure of texture feature code calculation of a palmprint image. Given a pixel of a palmprint image, we first calculate the pixel distance between the pixel and its eight neighboring points. Then, we select the two neighboring points with the two maximum pixel distances and encode the position of them into the texture feature code.

If two neighbor points have the same pixel distances with the center point, we select the smaller direction number to encode the texture feature. It is easy to check that is ranging from 1 to 62. Because the smallest direction number combination is {1, 2}, which is encoded into 1. The largest direction number combination is {8, 7}, which is encoded into 62. Figure 2 illustrates the basic idea of the robust texture feature extraction scheme.

It can be seen that the two neighbor points have the larger gray value differences with the center point than the other neighbors, so that they are more robust to small random noises. Therefore, our proposed texture feature extraction method can better represent the texture data of palmprint images.

### 3.2. Gradient Feature Extraction of Palmprint Images

**Figure 3:**
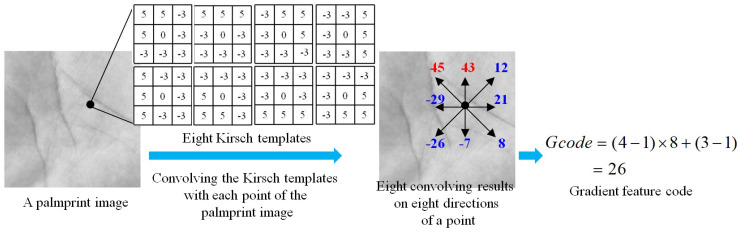
The basic procedure of gradient feature code calculation of a palmprint image. We first define eight Kirsch templates on eight different directions. Then, we calculate the convolution of the eight Kirsch templates with the palmprint image to obtain eights edge responses on eight directions. Finally, we encode the directions of the two templates with the maximum two edge responses into gradient feature code.

It is seen that a palmprint consists of rich textures, lines and wrinkles, which carry obvious edge information. To better represent there edge features, we employ the edge operators to exploit gradient features from palmprint images. In this paper, we use the simple yet effective Kirsch operator [13] to extract the gradient features. Specifically, we first employ eight Kirsch operators on eight different directions, and convolve them with the palmprint image to produce eight edge responses. Then, we sort the absolute values of these edge responses on eight directions. Finally, we select the direction numbers of the templates with the two maximum edge responses, and encode them as follows:

(4) $$Gcode=\left(p_{1}-1\right)\times 8+\left(p_{2}-1\right),$$

where $p_{1}$ and $p_{2}$ denotes direction number of the Kirsch templates with the top-two absolute edge responses, and they can be similarly calculated as (2) and (3). Gcode represents the gradient feature codes, which is also ranging from 1 to 62 as the Tcode. Figure 3 shows the main procedure of the gradient feature extraction and representation, where the direction number of templates is ordered as Figure 2.

### 3.3. Direction Feature Extraction of Palmprint Images

From the overview of palmprint descriptors, the directions have served as one of the most important and discriminative features and achieved promising effectiveness for palmprint recognition. Motivated by this, in this paper we also extract the direction features. In general, the common way of direction feature extraction first defines a group of direction-based templates, and then convolves these templates with the palmprint image to obtain the direction responses. Finally, it engineers different encoding schemes to convert the direction responses into the direction feature codes for palmprint representation and recognition.

There have been various direction-based templates that were used for direction feature extraction of palmprint, such as Gabor filters, Gaussian filters and MFRAT. Most existing studies [17] have shown that the Gabor filter-based templates can better characterize the direction characteristics, and thus can effectively extract the direction features of a palmprint. Due to this, in this paper, we also employ the Gabor filters as the direction-based templates to exploit the direction features of palmprint images. Specially, we first define twelve direction-based templates based on the real parts of the Gabor filters with the direction of $\theta_{j}=\left(j-1\right)\pi /N_{\theta }\;\left(j=1,\cdots ,N_{\theta }\right)$, where $N_{\theta }=12$ denotes the number of templates as well as the direction number. Suppose G(θ) represents the direction-based templates with direction θ, and *I* represents a palmprint image, we first calculate the direction responses of the palmprint by convolving these direction-based templates with the palmprint image as follows:

(5) $$c_{j}\left(x,y\right)=G\left(\theta_{j}\right)*I\left(x,y\right),\;\left(j=1,2,\ldots ,N_{\theta }\right),$$

where “*” represents the convolution symbol and $c_{j}$ represents the direction responses of the palmprint on the direction of $\theta_{j}$.

To better capture the discriminative direction features, we first select the direction with the maximum convolving response, referred to as $q_{1}$, which represents the most dominant direction features of the palmprint. Furthermore, we select the direction with the maximum direction response interval to its neighboring directions, where the direction response interval (DRI) of a direction can be calculated as follows:

(6) $$DRI_{j}\left(x,y\right)=|r_{j}\left(x,y\right)-r_{\varphi (j)}\left(x,y\right)|+|r_{j}\left(x,y\right)-r_{\phi (j)}\left(x,y\right)|,$$

where φ(j) and ϕ(j) denotes the two nearest neighboring directions of the direction $\theta_{j}$. φ(j) equals $N_{\theta }$ if j=1, and (j−1) otherwise. ϕ(j) equals 1 if $j=N_{\theta }$, and (j+1) otherwise. Therefore, the direction with the maximum direction response interval, referred to as $q_{2}$, can be selected as:

(7) $$q_{2}=\arg\max_{j}DRI_{j}\;\;\;\left\{j=1,2,\ldots ,N_{\theta }\right\}.$$

**Figure 4:**
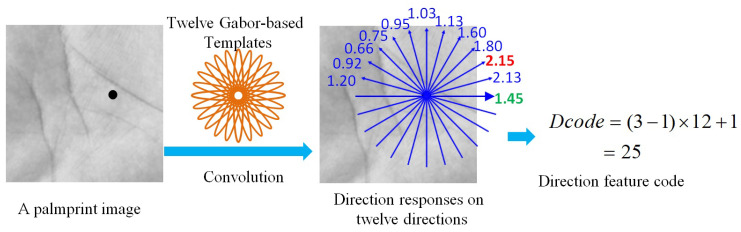
The basic procedure of direction feature code calculation of a palmprint image. We first define twelve direction feature templates based on Gabor filters with different directions. Then, we convolve these templates with the palmprint image to calculate the direction responses of twelve directions. Finally, we encode the direction with the maximum direction response and the maximum direction response interval into the direction feature code.

Previous studies have shown that the most dominant direction (i.e., $q_{1}$) has the competitive discriminative power. In addition, the direction with the maximum DRI has good reliability because it is hard to be affected by the neighboring directions. Due to these, we encode these two directions with the maximum convolving response and the maximum DRI as the direction features of the palmprint:

(8) $$Dcode\left(x,y\right)=\left(q_{1}-1\right)\times N_{\theta }+q_{2},$$

where Dcode represents the direction feature code. Figure 4 shows the basic idea of forming the direction feature code of a palmprint image.

### 3.4. Feature Matching Fusion

In general, feature level and matching score level are the most two effective schemes for multiple features fusion. It is note that different types of palmprint features have different discriminative power, and the feature-level fusion cannot better describe different features. Due to this, in this paper, we employ the matching score level fusion scheme to fuse our proposed triple-type palmprint features. Specifically, we first form block-wise histogram feature descriptors for triple types of palmprint features. Then, we fuse the matching score of triple types of feature descriptors by setting different weights for them. In the following, we detail the feature descriptor formation and weighted matching score-level fusion procedures.

It is well seen that different regions of a palmprint usually have obvious different vision characters. For example, some regions have more widely distributed lines and wrinkles, so that these regions contain more gradient and direction features. In order to overcome the small misalignment and achieve invariant palmprint representation, in this paper, we form block-wise histogram feature vectors as the feature descriptors for palmprint representation. Specifically, given a palmprint image, we first calculate the triple feature code maps of the palmprint image. Then, we divide there palmprint feature maps into non-overlapping blocks, the sizes of which are empirically set to 16×16 pixels. Third, we calculate the histograms of the texture feature codes, gradient feature codes and the direction feature codes for each block, respectively. Finally, we concatenate the block-wise feature code histograms into three feature vectors for triple type of features, respectively, so that triple-type feature descriptors, named as TFD, are obtained for a palmprint image.

Having obtained the triple types of feature descriptors of palmprint images, we use the simple yet effective Chi-square distance to calculate the similarity of two palmprint images. We first calculate the Chi-square distance for each pair of triple feature descriptors of the two compared palmprint images. Then, we fuse the triple Chi-square distances to form the final matching score of them as follows.

(9) $$S\left(u,v\right)=\sum_{i=1}^{3}w_{i}S_{i}\left(u,v\right),$$

where S(u,v) denotes the matching score of the compared feature descriptors, *u* and *v*, of two compared palmprint images. $u_{i}$ and $v_{i}\;\left(i=1,2,3\right)$ corresponding the feature descriptors of the texture, gradient, and direction features, respectively. $w_{i}>0\;\left(i=1,2,3\right)$ are the weighted parameters to make a suitable tradeoff the matching results of the triple feature descriptors, and $\sum_{i=1}^{3}w_{i}=1$. $S_{i}\;\left(i=1,2,3\right)$ represent the matching score calculated by using the Chi-square distance based on the *i* th feature descriptors, including the texture, gradient and direction feature descriptors, respectively, which can be calculated as follows:

(10) $$S_{i}\left(u,v\right)=\sum_{k=1}^{N_{i}}\frac{{\left(u_{i,k}-v_{i,k}\right)}^{2}}{u_{i,k}+v_{i,k}},$$

where $u_{i,k}$ and $v_{i,k}$ denotes *k* th bin of the $u_{i}$ and $v_{i}$, respectively. $N_{i}$ is the feature size of the $u_{i}$ and $v_{i}$ feature descriptor. Therefore, a smaller matching score of S(u,v) means of higher similarity of the two compared palmprint images. By doing this, the similarity of two palmprint images can be easily calculated for palmprint recognition.

## 4. Experiment

In this section, we first introduce three widely used palmprint image databases for our experiments, including the CASIA, IITD and GPDS palmprint image databases. Then, we conduct palmprint verification and identification experiments and analyze the experimental results. Finally, we analyze the computational time cost of the proposed method. All experiments are conducted under a platform including a PC with a double-core Intel(R) i7-7700 (3.60 GHz) CPU and 16 GB RAM.

### 4.1. Databases

The CASIA palmprint image database [45] consists of 5502 palmprint images captured with normal camera from both the left and right palms of 312 subjects. Each palm provided about 8 to 17 samples of the whole hands. Therefore, the CASIA database contains 612 different classes of palmprint images, so that the IITD database consists of 460 different classes of palmprint images because the samples of the left and right hands are considered as different classes. In this experiment, we used the Palmcode method to extract the ROIs of the palmprint images and resized them into 128 × 128 pixels.

The IITD palmprint image database [46] consists of 2601 contactless palmprint images captured from 230 individuals of the both left and right hands, each of which provided 5 or 6 images. Therefore, the IITD database consists of 460 different classes of palmprint images because the samples of the left and right hands are considered as different classes. All the palmprint images were captured by using common camera in a box, so that the hand poses such as rotation and translation are different. The ROIs with the sizes of 128 × 128 pixels have already been cropped and available in the database.

The GPDS palmprint image database [47] consists of 1000 contactless palmprint images of 100 subjects of the right hands, each of which contains 10 palmprint images as well as theirs ROIs. In the experiments, all ROIs of the GPDS databases were resized into 128 × 128 pixels.

**Figure 5:**
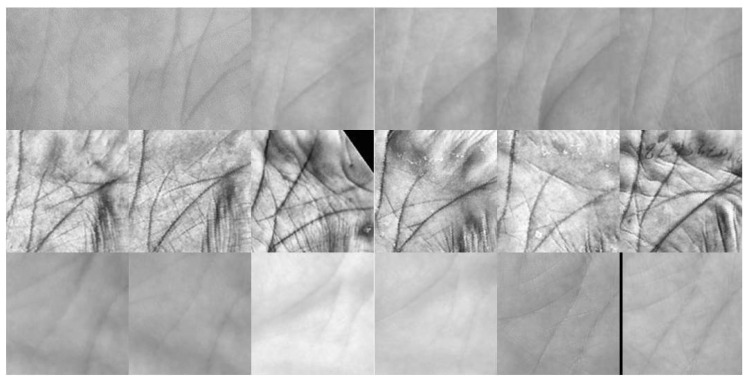
The typical palmprint image samples selected from the CASIA, IITD and GPDS databases, respectively, corresponding to the first to third lines.

Figure 5 presented some typical samples of the palmprint images selected from the CASIA, IITD and GPDS databases. It is seen that that different samples of different databases shows very different characteristics.

### 4.2. Palmprint Verification Results

In this subsection, we conduct palmprint verification experiments on the CASIA, IITD and GPDS databases. In general, palmprint verification is to compare a query image with a labeled image to verify whether the query image is from the same individual as the labeled image or not. In this experiment, we compare each pair of palmprint images from the same database. A compare is named as genuine matching if the two compared palmprint image are from the same palm, and otherwise called as an impostor matching. Then, we calculate the false acceptance rate (FAR) and genuine acceptance rate (GAR) [7] on each database to evaluate the proposed method. For a fair evaluation, we also implement several state-of-the-art palmprint presentation and recognition methods, such as competitive code [23], ordinal code [30], HOL [29], DoN [28], E-BOCV [25], DRCC [39] and ALDC [40] methods, and compared them with our proposed method. For our proposed TFD method, we empirically set weighted parameters: $w_{1}$, $w_{2}$ and $w_{3}$ to 0.1, 0.1 and 0.8, respectively. We will comparatively analyze the settings of these parameters in Section 4.4.

**Figure 6:**
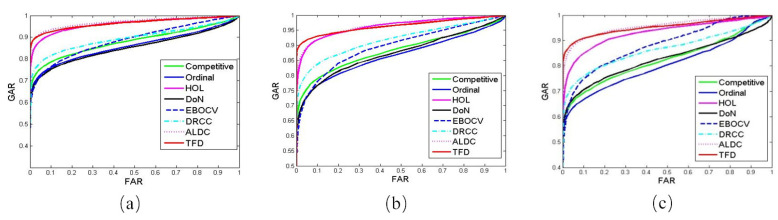
The ROCs of different methods on the (**a**) CASIA, (**b**) IITD, and (**c**) GPDS databases.

Figure 6 shows the ROC curves, i.e., FAR versus GAR, of our proposed method and the other compared methods. We can see that our proposed method achieves comparable and even better GAR than all the seven compared methods against the same FAR. This is because our proposed method can extensively exploit multiple features of palmprint images, which can provide more informative information for palmprint representation. By contrast, the conventional heuristic palmprint recognition methods such as competitive code, E-BOCV, DoN, only exploit the direction features, which cannot complete represent the texture and gradient information of palmprint images. In addition, we see that the proposed method achieves a slight better performance on the IITD database than that on the CASIA and GPDS databases. The possible reason is that the palmprint images of the IITD consists of more distinct line patterns than the samples of the CASIA and GPDS databases. These distinct patterns can provide more discriminative gradient-based and direction-based features, such that a better recognition accuracy rate can be obtained on the IITD database.

### 4.3. Palmprint Identification Results

Different palmprint verification, palmprint identification is to compare a query sample with a group of labeled samples and aims to identify the label of the query sample. In the palmprint identification experiment, for each database, we first randomly select *n* palmprint images per each palm to form a gallery sample set, and use the rest as the query samples, where *n* is set to 1 to 4, respectively. Then, we calculate the rank-one identification accuracy rates of the proposed method and the conventional representative methods. Specifically, we compare the proposed method with representative palmprint recognition methods including the competitive code, ordinal code, HOL, DoN, EBOCV, DRCC and ALDC. Moreover, the representative texture descriptors such as LBP [48] and LDP [49] were also implemented and compared. For fair comparisons, all methods were repeated 10 times and the average identification accuracy rates were reported. Table 1 tabulates the average rand-one identification results of the different methods on the CASIA, IITD, and GPDS databases.

From Table 1, we can see that our proposed method consistently outperforms the nine compared by achieving obviously higher rand-one identification rates than the others. Specifically, our proposed method achieves much better accuracy rates than the direction-based palmprint recognition methods such as competitive code, DoN and DRCC. The possible reason is that, compared with the direction-based methods, our proposed method not only extracts the direction-type features but also exploits the texture-type and gradient-type features, which can provide more informative and discriminative features over the direction features, so that higher identification accuracy rates can be obtained. Moreover, our proposed method significantly improves the identification accuracy rates over the LBP and LDP methods. This is because the LBP and LDP only describe the single-type features such as texture-based and edge-based features. By contrast, our proposed method can extract more direction-based and gradient-based features, which have shown promising discriminative power for palmprint recognition, such as a better recognition accuracy rate can be obtained.

### 4.4. Parameter Analysis

**Figure 7:**
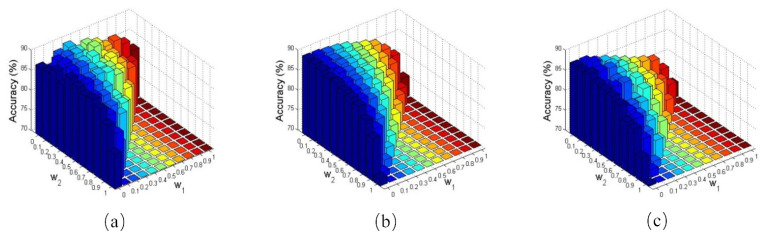
The average identification accuracy rates of the proposed method versus different values of weighted parameters on the (**a**) CASIA, (**b**) IITD, and (**c**) GPDS databases.

Our proposed method extracts triple types of palmprint features and fuse them in a weighted matching score level fusion scheme. To evaluate the importance and discriminative power of different types of features, we set different values of weighted parameters and compare the identification performance of the proposed method. It is impractical to enumerate all possible values for these parameters. Due to this, in this experiment, we first set $w_{1}$ with the values ranging from 0 to 1 with the interval of 0.1, and set $w_{2}$ from 0 to $1-w_{1}$ with the interval of 0.1. Accordingly, we set the $w_{3}$ as: $w_{3}=1-w_{1}-w_{2}$. After that, we perform palmprint identification with the proposed method on the CASIA, IITD and GPDS databases, where one palmprint image per palm was randomly selected as the gallery sample. Figure 7 describes the comparative accuracy rates of the proposed method versus different values of the parameters.

We can see from Figure 7 that the proposed method usually performs the best when $w_{1}$ and $w_{2}$ are set to around 0.1 to 0.3, and accordingly $w_{3}$ is set to about 0.7 to 0.8. This demonstrates that the direction features of palmprint images usually convey more discriminative features than the other two types of features. This is consistent with the existing studies that the state-of-the-art palmprint methods usually extract the direction features for palmprint recognition. In addition, the experimental results also clearly show that, by combining the triple types of features with set suitable weights, the proposed method can achieve obvious better recognition performance than the single-type feature representation, demonstrating the effectiveness of the proposed method.

### 4.5. Computational Time Analysis

To evaluate the efficiency of feature extraction of our proposed method, in this subsection, we calculate the time cost of our proposed method for feature extraction. Moreover, we compared the computational cost of the proposed method with state-of-the-art feature extraction methods such as competitive code, ordinal code, EBOCV, DoN and DRCC methods. For a fair evaluation and comparison, we extract the features of 100 palmprint images based on different methods and report the average time taken of feature extraction for a palmprint image. Table 2 summarizes the average feature extraction time taken of different methods. We can see that the proposed method achieves a slight higher time cost than the compared methods. The possible reason is that our proposed method extracts triple times of features than the other feature descriptors. It is worth noting that our proposed method takes about 0.05 s for feature extraction for a palmprint image, which is acceptable for practical applications. Therefore, since our proposed method can significantly improve the recognition accuracy over the existing methods, our proposed TFD method can make a good tradeoff when the recognition effectiveness and efficiency are both concerned.

## 5. Conclusions

In this paper, we propose a triple-type feature descriptor for palmprint recognition. To completely exploit the discriminative features, our proposed method respectively extracts the texture-type, gradient-type and direction-type features, which are the most important components of a palmprint image. Then, we use the simple and effective matching score level fusion to combine triple-type features for palmprint matching. Extensive experiments on three challenging palmprint databases clearly show that our proposed method outperforms previous palmprint descriptors. For future work, we will further explore other types of hand-crafted features to further improve the performance for palmprint recognition.

## Figures and Tables

**Table 1:** The average rank-one identification accuracies of different methods on the CASIA, IITD, and GPDS databases.

	LBP	LDP	Competitive	Ordinal	HOL	DoN	EBOCV	DRCC	ALDC	TFD
CASIA	48.36	52.39	55.21	47.26	83.03	59.99	60.50	58.79	86.16	88.55
	60.83	63.47	66.49	67.66	88.37	74.25	75.55	70.24	92.03	94.35
	71.21	72.12	79.45	75.92	92.45	80.03	82.83	78.59	93.65	95.55
	72.30	72.65	79.27	73.26	94.87	80.37	84.06	81.45	94.64	96.88
IITD	43.64	43.87	45.92	42.25	84.88	60.71	60.73	55.81	85.07	89.04
	58.33	59.62	65.16	58.77	93.19	68.12	74.31	73.44	93.53	94.97
	62.12	62.87	72.25	70.73	95.12	73.43	84.10	80.14	96.15	96.83
	64.56	64.44	79.79	76.43	96.80	80.69	87.96	85.04	97.00	97.47
GPDS	50.23	52.74	61.73	56.18	79.35	61.16	60.56	47.77	85.53	85.55
	66.12	68.33	75.88	74.68	91.37	75.78	75.60	68.70	92.85	95.30
	69.43	70.20	80.03	82.17	93.31	80.13	84.71	75.22	95.05	96.34
	70.75	70.87	86.03	85.53	96.10	85.71	87.16	81.23	97.70	98.33

**Table 2:** The average time taken (in second) of different methods for extracting the features of a palmprint image.

Methods	Feature Extraction Time Taken
Competitive	0.0136
Ordinal	0.0152
EBOCV	0.0201
DoN	0.0102
DRCC	0.21
TFD	0.0225

## Data Availability

No new data were created or analyzed in this study. Data sharing is not applicable to this article.
